# Brugada Phenocopy Type 1 Secondary to Synthetic Cannabinoids

**DOI:** 10.1155/2020/9425860

**Published:** 2020-06-23

**Authors:** Mohammad Amro, Kelechukwu U. Okoro, Kanaan Mansoor, Ahmed Amro, Paul Okhumale

**Affiliations:** ^1^School of Medicine, Misr University for Science and Technology, Cairo, Egypt; ^2^Marshall University School of Medicine, Huntington, WV, USA

## Abstract

Brugada phenocopies (BrP) are clinical entities that have EKG tracings similar to the congenital Brugada syndrome (BrS) but without ventricular tachyarrhythmias or sudden cardiac death. BrP is caused by various factors such as metabolic disturbances (electrolyte imbalance), drugs, mechanical compression of the mediastinum, and inflammatory conditions such as myocarditis or pericarditis. We present a very rare case of a young patient who had a Brugada phenocopy Type 1 suspected to be secondary to synthetic cannabinoids.

## 1. Introduction

BrS is an autosomal dominant genetic disorder that is associated with sudden cardiac death (SCD) secondary to ventricular tachyarrhythmia in the setting of a structurally normal heart [1]. It is characterized by three morphologic phenotypes. Type 1 is referred to as the coved pattern, Type 2 is referred to as the saddle-back pattern, and Type 3 demonstrates the characteristics of either Type 1 and/or 2 without meeting all the criteria for either Type 1 or 2.

BrP are clinical conditions that demonstrates classical Brugada pattern on ECG but do not lead to sudden cardiac death (SCD) from ventricular tachyarrhythmia [2]. Distinguishing between BrS and BrP is most important as the management is different for both diagnoses. The primary objective of BrS is prevention of SCD; thus, patients usually receive an implantable cardioverter defibrillator (ICD) once diagnosis is made. In patients with BrP, implantation of ICD is not indicated as these patients do not go onto develop SCD. Also, ECG changes in patients diagnosed with BrP are transient and secondary to an underlying clinical condition. Once the underlying condition is rectified, characteristic Brugada ECG changes resolve.

## 2. Case Presentation

A 33-year-old Caucasian gentleman was admitted to our facility after he was found unresponsive by his girlfriend after an unknown prolonged absence. The girlfriend could not arouse the patient despite multiple attempts. Paramedics arrived on the scene and administered 6 mg of Naloxone which was successful in resuscitating the patient. Upon initial interrogation, the patient reported that he had snorted heroin. There was also report that he has been using a form of impure heroin colloquially known as “Black Tar Heroin” due to its characteristic appearance which is similar to roofing tar. Despite this, his toxicology screen was negative for heroin, but it did return with a positive result for synthetic cannabinoids.

**Figure 1:**
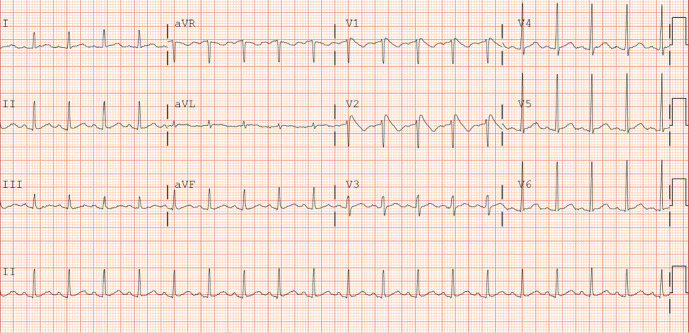
ECG on admission demonstrating characteristic Type 1 coved shaped ST segment elevation in leads V1 and V2.

**Figure 2:**
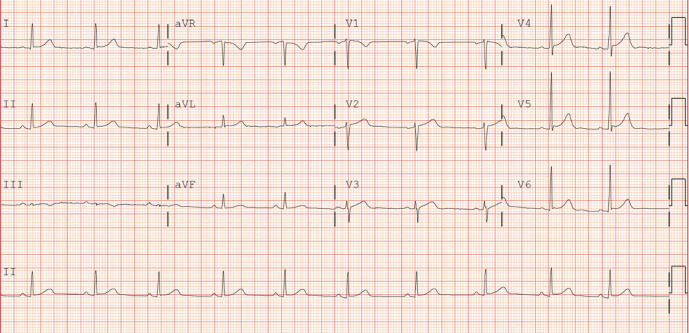
ECG two days after admission demonstrating absence of characteristic Brugada pattern.

Serum chemistry demonstrated lactic acid of 7.2 mmol/L, bicarbonate 16 mmol/L, blood glucose 271 mg/dL, BUN 17 mg/dL, and serum creatinine 2.3 mg/dL. Initial troponin was 0.045 ng/dL but trended upwards to 0.926 ng/dL. Creatinine kinase was 506 u/L and peaked at 7804 u/L before trending downwards. The patient's initial EKG demonstrated coved ST elevations in V1 and V2 which are characteristic changes consistent with BrS Type 1 (Figure 1). Repeat EKG after 1 day of admission demonstrated resolution of coved ST segment elevation (Figure 2). Electrophysiology (EP) service was consulted for implantation of ICD. The patient was evaluated and admitted for several occasions previously of loss of consciousness associated with drug overdose albeit he was not entirely certain. Due to reported history of multiple unclear episodes of loss of consciousness, we elected to proceed with EP study. Moving the EKG leads one rib space up which also failed to demonstrate the previously appreciated ECG changes. We proceeded with the Procainamide challenge. We administered Procainamide at 100 mg/min for 10 minutes till we achieved a maximum dose of 1 gm while continuously monitoring the patient's rhythm. We were unable to elicit symptoms or ECG changes consistent with BrS. As a result, the patient was diagnosed with BrP attributable to drugs of abuse which was synthetic cannabinoids based on toxicology screen which showed positive result for synthetic cannabinoids. The patient was diagnosed with BrP and discharged home.

## 3. Discussion

BrS is an autosomal dominant genetic channelopathy that is associated with sudden cardiac death (SCD) secondary to ventricular tachyarrhythmia in the setting of a structurally normal heart [1–3]. It is inherited in an autosomal dominant pattern with variable penetrance with male predominance [4, 5]. The gene most associated with BrS is SCN5A which encodes for a cardiac sodium channel [5]. Mutations in this channel lead to unopposed potassium current in the RV epicardium through the I_to_ potassium channel. BrS is characterized by characteristic appearance on the ST segment and T wave in the anterior precordial leads [5, 6]. BrS is subclassified into three types depending on phenotypic appearance on the EKG (Table 1). Type 1 is referred to as the coved pattern, Type 2 is referred to as the saddle-back pattern, while Type 3 is a combination of both Types 1 and 2 without satisfying the criteria for either Type 1 or 2 [2–4, 7].

BrS ECG alterations can be dynamic, i.e., characteristic ECG patterns are absent at baseline but are “unmasked” due to derangements in homeostasis such as pyrexia and electrolyte abnormalities. Similar to BrS, BrP is also considered multifactorial [8, 9]. Patients with BrS are usually asymptomatic until they present with syncope or SCD [5]. Ventricular arrhythmia associated with BrS tends to occur nocturnally, in the setting of pyrexia particularly with children, or at rest especially after a large meal [3]. Adults usually succumb to either polymorphic VT or VF while the predominant arrhythmia in children is monomorphic VT. There is no medication that is indicated for management of BrS; rather individuals that receive a diagnosis of BrS require implantation ICD to prevent SCD.

BrP on the other hand is described as a selection of clinical disorders that demonstrate classic BrS ECG phenotypical appearance but lack the propensity to cause ventricular tachyarrhythmia and sudden death [2, 4, 10]. These disorders are classified by their etiological category which include metabolic imbalance, mechanical compression, ECG modulation, myocardial or pericardial disease, ischemia or pulmonary embolism, and miscellaneous causes.

**Figure 3:**
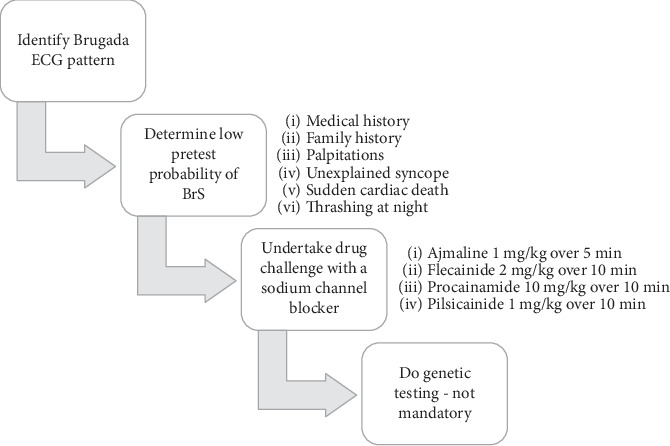
International Registry of Brugada Phenocopies proposed algorithm for appropriate diagnosis of Brugada phenocopies.

It is important to properly identify unmasked BrS vs. BrP in patient who develops characteristic Brugada ECG pattern. The International Registry of Brugada Phenocopies have proposed an algorithm which allows for appropriate diagnosis of BrP (Figure 3) [11]. Of most importance in the algorithm is the provocation/challenge test with sodium channel blockers [1, 5]. These medications act by blocking predominantly sodium currents thus increasing the already-present ionic imbalance. This test should only be performed in a setting where continuous ECG monitoring is available and in the presence of health care providers who can provide management in the setting of an emergency. A positive result is connoted by the depiction of Brugada pattern [3]. The inability to demonstrate Brugada pattern constitutes a negative result and a diagnosis of BrP. The appearance of QRS widening, occurrence of frequent PVCs, or complex ventricular arrhythmias should lead to the cessation of the challenge. Isoprenaline infusion may be utilized to counteract the development of ventricular arrhythmias [3].

Once diagnosed, BrP is classified in the same vein as BrS, i.e., depending on the phenotypic appearance on ECG. A further subclassification is designated to BrP depending on the criteria met in the aforementioned algorithm. Class A represents cases in which all criteria were met, class B includes cases in which not all criteria are met but BrP is highly suspected, and class C are cases with unjustified provocative testing [1, 12]. The utilization of the algorithm is most important as it has been demonstrated that an experienced cardiologist cannot discern between BrP and BrS by ECG alone [1, 12, 13].

Our patient presented to the ED after drug overdose. It is unclear what he may have consumed as he was not forthcoming with details surrounding the events before his loss of consciousness. His toxicology screen demonstrated the presence of synthetic cannabinoids despite the fact he reported use of heroin multiple times in the past.

Drugs of abuse such as cannabinoids, heroin, and cocaine have been associated with BrP [14]. Changes in the ECG secondary to heroin such as QTc prolongation, torsades de pointes, and bradyarrhythmia are also well documented [15]. Ramsaroop et al. described a patient who required Naloxone to be resuscitated with positive toxicology screen for alcohol and opiates [15]. Ghovanloo et al. demonstrated that cannabinoids have a potential inhibitory effect on sodium channels under certain circumstances [16]. However, synthetic cannabinoids effect on sodium channels is not described in literature; also, our review of literature failed to demonstrate any reports or cases of synthetic cannabinoids leading to unmasked BrS or BrP.

The patient's EKG demonstrated Type 1 Brugada pattern which spontaneously resolved after several hours. Our patient also reported several episodes of loss of consciousness in the past. He was unclear what the etiology of his syncope was, but he believed it to be secondary to drug abuse although he was uncertain. As a result, we had to entertain the idea of a diagnosis of unmasked BrS vs. BrP. We proceeded with sodium channel provocation/challenge test. Procainamide was chosen as the challenge drug due to availability. Once it was ensured that all affairs were in order, we proceeded with the test. There was no QT prolongation, ventricular arrhythmia, PVC, or development of Type 1 Brugada pattern. The patient remained asymptomatic throughout the entirety of the examination.

## 4. Conclusion

Clinicians should be aware of the differentiation between BrS and BrP. Both conditions are distinct from each other as thus management is different. Early recognition of the presence of each disease can prevent misdiagnosis and inappropriate treatment.

## Figures and Tables

**Table 1:** Patterns of ST abnormalities in leads V1-V3.

Feature	Type 1	Type 2	Type 3
J wave amplitude	≥2 mm	≥2 mm	≥2 mm
T wave	Negative	Positive or biphasic	Negative, positive, or biphasic
ST-T configuration	Coved	Saddle-back	Coved or saddle-back
Terminal portion of ST segment	Gradually descending	Elevated ≥1 mm	Gradually descending or elevated ≥1 mm
